# High-energy-resolution off-resonant spectroscopy with self-seeded x-ray free-electron laser pulses

**DOI:** 10.1063/4.0000243

**Published:** 2024-03-26

**Authors:** Jang Hyeob Sohn, Gyeongbo Kang, Tae-Kyu Choi, Gyusang Lee, Changhoo Lee, Sae Hwan Chun, Jaeku Park, Dongbin Shin, Byoung-Ick Cho

**Affiliations:** 1 Department of Physics and Photon Science, Gwangju Institute of Science and Technology (GIST), Gwangju 61005, South Korea; 2 XFEL Division, Pohang Accelerator Laboratory, POSTECH, Pohang, Gyeongbuk 37673, South Korea; 3 Center for Relativistic Laser Science, Institute for Basic Science (IBS), Gwangju 61005, South Korea; 4 Max Planck Institute for the Structure and Dynamics of Matter and Center for Free Electron Laser Science, 22761 Hamburg, Germany

## Abstract

This paper presents the implementation of high-energy-resolution off-resonant spectroscopy (HEROS) measurements using self-seeded x-ray free-electron laser (XFEL) pulses. This study systematically investigated XFEL conditions, including photon energy and accumulated shot numbers, to optimize the measurement efficiency for copper foil samples near the *K*-edge. The x-ray absorption spectra reconstructed using HEROS were compared with those derived from fluorescence-yield measurements. The HEROS-based spectra exhibited consistent line shapes independent of the sample thickness. The potential application of HEROS to high-temperature copper was also explored. HEROS offers distinct advantages including scan-free measurement of x-ray absorption spectra with reduced core-hole lifetime broadening and self-absorption effects. Using self-seeded XFEL pulses, HEROS facilitates single-shot-based pump–probe measurements to investigate the ultrafast dynamics in various materials and diverse conditions.

## I. INTRODUCTION

Over the past decade, x-ray free-electron lasers (XFEL) have substantially broadened the scope of various experimental domains by delivering femtosecond x-ray pulses with exceptionally high brightness and spatial coherence.^1–5^ For example, it enables the exploration of the extraordinary and transient properties of matter under extreme conditions, such as warm and hot dense matter characterized by elevated temperatures and pressures. The capability to conduct single-shot measurements with a femtosecond XFEL pulse offers distinct advantages when probing matter that undergoes irreversible processes. Scientists have leveraged spectroscopic techniques such as x-ray emission or absorption spectroscopy (XES or XAS) to make substantial strides in examining the structures of extreme matter and plasma, and tracing their dynamic evolution.^6–13^ There is growing interest in employing more advanced techniques on XFEL platforms, amplifying the investigation of such matter at extremely high pressures and temperatures.

High-energy-resolution off-resonant spectroscopy (HEROS) is a powerful technique for observing inelastic x-ray scattering spectra in off-resonant energy regions using a high-resolution energy-dispersive spectrometer. HEROS provides detailed information on the electronic structures of matter with reduced core-hole lifetime broadening and minimized dependence on the penetration depth of incident photon energy (incident beam self-absorption).^14–23^ However, its application to the study of matter under extreme conditions has been limited. Recent efforts by Humphries *et al.* demonstrated the capability of diagnosing temperatures and the *K*-shell absorption edge of warm dense nickel by measuring inelastic x-ray scattering under off-resonant conditions using XFEL pulses.^24^ Nonetheless, the broad bandwidth of self-amplified spontaneous emission (SASE) pulses and the low instrumental resolution pose measurement uncertainties that impede comprehensive investigations.

This paper presents HEROS measurements using self-seeded XFEL pulses and an energy-dispersive von Hamos spectrometer as a novel approach for acquiring comprehensive information on the unoccupied density of states (DOS). The HEROS spectrum of copper was systematically measured and analyzed at various photon energies. The x-ray absorptions extracted from the HEROS measurements were compared with fluorescence-based XAS measurements. The calculated HEROS spectra of the laser-heated copper at elevated temperatures are also presented. These investigations highlighted the capability of HEROS with a self-seeded XFEL to discern detailed information on the transient electronic structures of matter under extreme conditions.

## II. THEORETICAL BACKGROUND

**FIG. 1.**
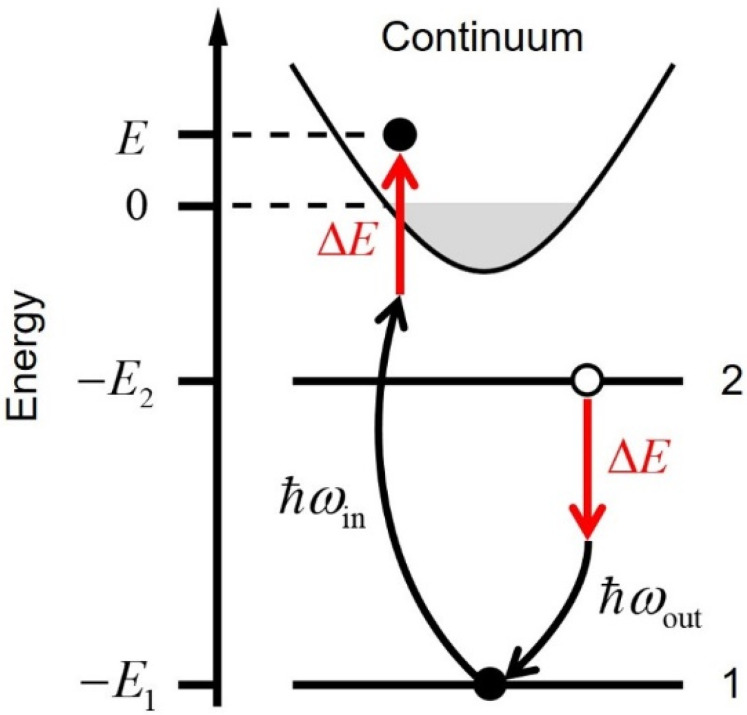
A schematic illustration of an inelastic x-ray scattering process relevant to the HEROS.

HEROS is a high-resolution spectroscopic technique used to measure the inelastic x-ray scattering in the off-resonant regime, where the incident photon energy falls below the ionization threshold of a specific atomic level. Figure 1 schematically depicts the inelastic x-ray scattering process relevant to HEROS. Despite photoexcitations occurring off resonance, the emission of x-ray photons becomes feasible due to excited electrons receiving additional energies (Δ*E*) from the quasi-simultaneous decay process. This unique interplay results in the HEROS spectra manifesting in a lower energy domain than in the original decay process. This is a one-step process in which excitation and de-excitation occur simultaneously. This coherence endows HEROS with the capability of obtaining highly resolved spectra unaffected by interference from the lifetime broadening of the inner shell.^18,22,25^

HEROS is described by the Tulkki–Åberg equation, which represents the differential cross section of resonant inelastic x-ray scattering (RIXS):^26^

$$\frac{d\sigma \left(\omega_{\text{in}}\right)}{d\omega_{\text{out}}}\propto \frac{\omega_{\text{out}}}{\omega_{\text{in}}}\int \frac{g_{2-1}\left(dg_{1}/d\omega \right)\left(E_{1}-E_{2}\right)\left(E_{1}+\hbar \omega \right)}{{\left(E_{1}+\hbar \omega -\hbar \omega_{\text{in}}\right)}^{2}+\Gamma_{1}^{2}/4}\times \frac{\Gamma_{2}/2\pi }{{\left(E_{2}+\hbar \omega +\hbar \omega_{\text{out}}-\hbar \omega_{\text{in}}\right)}^{2}+\Gamma_{2}^{2}/4}d\omega ,$$ (1)

where $\hbar \omega_{\text{in}}$ and $\hbar \omega_{\text{out}}$ are the incoming and outgoing photon energies, respectively. *E*_1_ and *E*_2_ represent the absolute values of the energies at Levels 1 and 2, respectively. Γ_1_ and Γ_2_ denote the corresponding broadenings of the levels. $g_{2-1}$ is the oscillator strength for the 2–1 radiative transition, and $dg_{1}/d\omega$ is the oscillator strength distribution for electron excitations from level 1.

By employing monochromatic incident x-ray sources, the scattering cross section on the left-hand side of Eq. (1) can be replaced with the intensity of the emitted photons. Assuming that Γ_2_ is negligible, the second Lorentzian function representing the density function of level 2 is also replaced by the Dirac delta function.^21,26^ Assuming that the oscillator strength distribution of excited electrons is proportional to the unoccupied DOS, the intensities of the HEROS spectra can be reformulated as follows:

$$I_{\text{HEROS}}\left(\omega_{\text{out}}\right)\propto g_{2-1}\rho \left(E\right){\left|M\left(E\right)\right|}^{2}\left[1-f\left(E;T\right)\right]\times \frac{\omega_{\text{out}}}{\omega_{\text{in}}}\frac{\left(E_{1}-E_{2}\right)\left(\hbar \omega_{\text{in}}-\hbar \omega_{\text{out}}+E_{1}-E_{2}\right)}{{\left[\hbar \omega_{\text{out}}-\left(E_{1}-E_{2}\right)\right]}^{2}+\Gamma_{1}^{2}/4},$$ (2)

where *ρ*(*E*) is the DOS for the continuum and *M*(*E*) are the dipole matrix elements for 1*s*-to-continuum transitions. *f*(*E;T*) is the Fermi–Dirac distribution at temperature *T*. $E=\hbar \omega_{\text{in}}-\hbar \omega_{\text{out}}-E_{2}$ is the final kinetic energy of the excited electron.

Equation (2) illuminates the role of the accessible unoccupied DOS, which is also directly coupled to the x-ray absorption cross sections, in shaping the HEROS spectrum. This inherent connection allowed us to derive the HEROS spectrum based on the DOS data or x-ray absorption spectrum. Reciprocally, the HEROS measurements provide an avenue for implementing scanning-free XAS using monochromatic x-ray sources. This feature is particularly advantageous when considering the limited availability of XFEL facilities and positioning the scanning-free method as an efficient and strategic data-acquisition technique. In addition, in HEROS measurements, both incoming and outgoing photon energies are fixed below the absorption edge, minimizing spectral distortions due to energy-dependent absorption by the sample.^17,18,21^ These combined characteristics endow HEROS with unique advantages, enabling the efficient and accurate investigation of electronic structures across diverse experimental conditions.

## III. MEASUREMENT AND RESULT

### A. Experimental details

The experiment was performed at the femtosecond x-ray scattering end station of the PAL–XFEL. The XFEL was operated in the self-seeded mode. A seed pulse was filtered by a 100-*μ*m-thick diamond crystal after passing through upstream undulators and then amplified in downstream undulators.^27–29^ The x-ray pulse was filtered using a double-crystal monochromator (DCM) to suppress the SASE background.^30^ The self-seeded pulses had photon energies tunable in the range of 8965–9000 eV with a spectral bandwidth (0.5 eV) three times narrower than the SASE pulses filtered by the DCM (1.5 eV). The average pulse energy was 0.75 ± 0.34 mJ, corresponding to 3–7 × 10^11^ photons/pulse, six times higher than the SASE pulses within the same bandwidth. The pulse duration was 30 fs at the full width at half maximum (FWHM). The self-seeded pulses were focused onto a target by beryllium compound refractive lenses to a diameter of 30 *μ*m FWHM. The pulses were delivered at a repetition rate of 60 Hz with an incidence angle of 37° from the target normal direction.

Cu foils, either 5- or 25-*μ*m-thick, served as targets and were rastered for each shot to prevent probing of melted Cu, as a few self-seeded x-ray shots had caused damage to the foils. However, the x-ray shot intensities were not set too high to distort the HEROS spectra, thus affecting only the signal-to-noise ratios (SNRs). The emitted x-ray photons were analyzed using a von Hamos spectrometer consisting of two cylindrically bent Si(440) crystals with a 500-mm radius of curvature and a JUNGFRAU detector positioned on the Rowland circle in the vertical diffraction plane. The crystals were aligned for Cu *Kα*_1_ emissions at a Bragg angle of 53° and calibrated to Cu *Kα*_1_ and *Kα*_2_ emission lines. The ideal energy resolution in this measurement was 0.7–0.8 eV for the spectral range 7900–8100 eV, primarily determined by the bandwidth of the self-seeded beam and the angular divergence caused by the finite size of the x-ray source and detector pixel (75 *μ*m). However, the actual resolution was estimated to be 1.3–1.4 eV, owing to either the x-ray beam positional jitter or the non-uniform spatial distribution of incident photon energies.^30,31^ Furthermore, details of the self-seeded beam and von Hamos spectrometer at the PAL–XFEL are presented in Ref. 30.

### B. HEROS spectra of copper foil

**FIG. 2.**
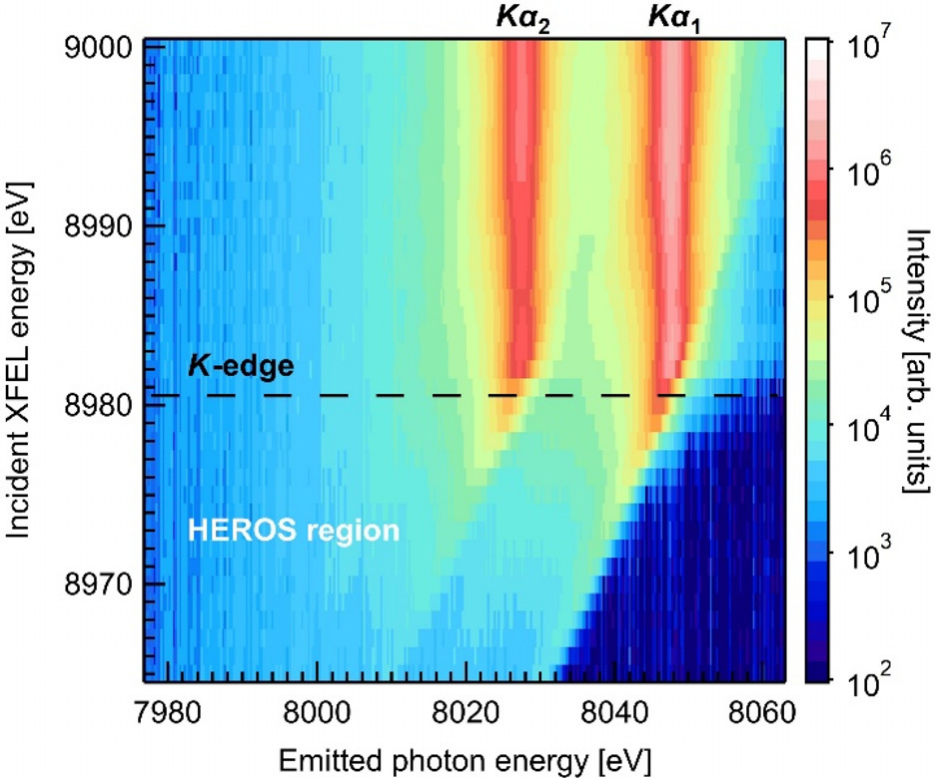
The intensity map of emitted x-ray photons from 5-*μ*m-thick Cu foils.

Figure 2 shows the intensity map of emitted x-ray photons from 5-*μ*m-thick Cu foils above and below the *K*-absorption edge. The XFEL photon energy was scanned at a step size of 1 eV, and 3000 images were averaged into a line shape for each XFEL energy. Above the *K*-edge (dotted line; 8980.5 eV^32^), the prominent *Kα*_1_ and *Kα*_2_ emission lines emerge. Below the edge, where the photoionization processes are suppressed, distinctive inelastic x-ray scattering signals, i.e., the HEROS spectra, are recorded in the off-resonant region. The peak energies of the HEROS spectra exhibit down-shifts according to the incident XFEL energies, different from the *Kα* fluorescence.

**FIG. 3.**
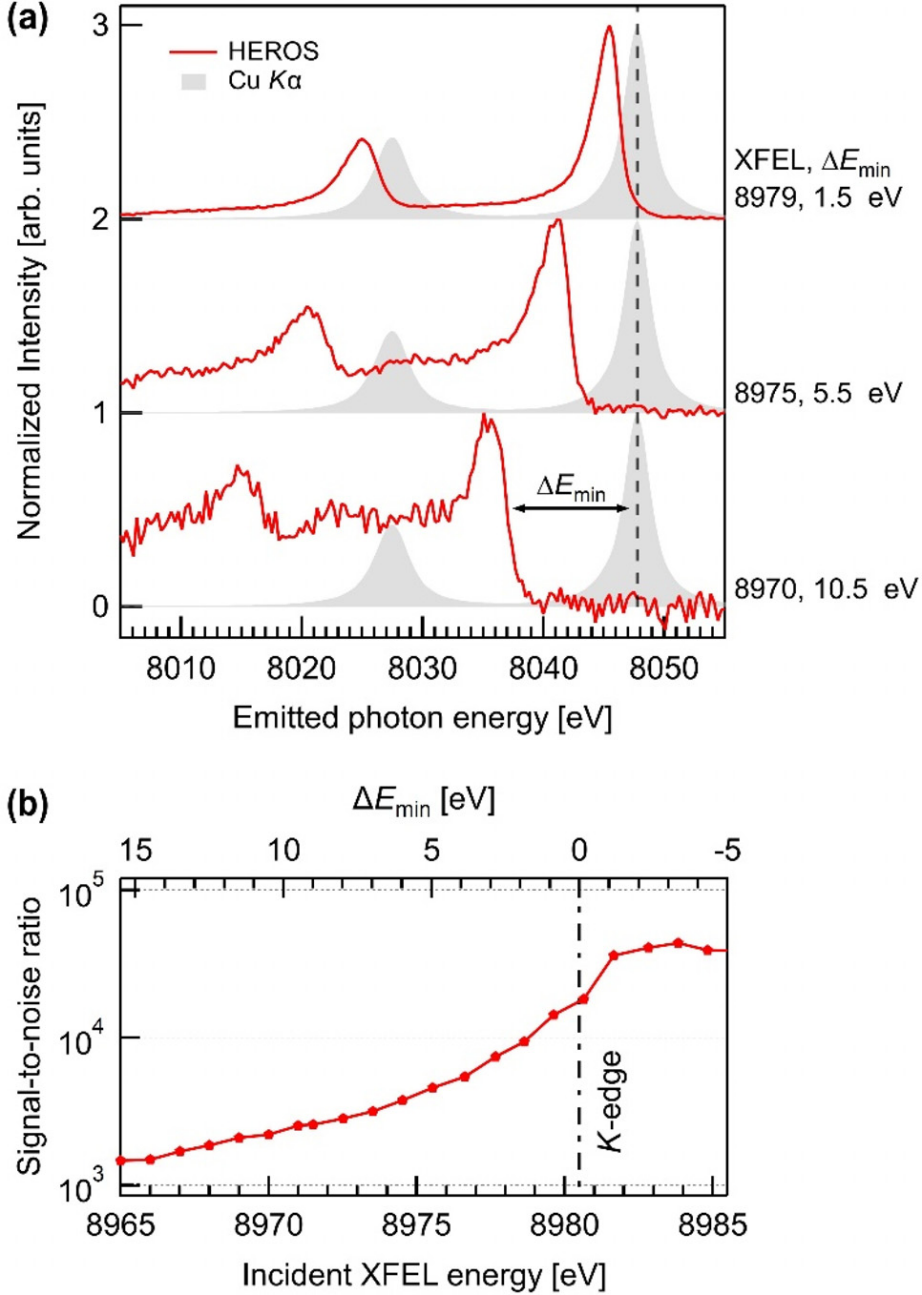
(a) The HEROS spectra at different XFEL photon energies of 8970, 8975, and 8979 eV. Δ*E*_min_ indicates the energy difference between the *Kα*_1_ maximum and the cutoff edge of a HEROS spectrum. The spectra are displayed with a vertical offset for clarity. (b) The SNRs of the averaged spectra at various XFEL energy (bottom axis) and the corresponding Δ*E*_min_ (top axis).

Figure 3(a) presents the HEROS spectra averaged for 3000 x-ray shots at three XFEL energies (8979, 8975, and 8970 eV) alongside the *Kα* emission spectra. Each spectrum exhibited a similar line shape comprising scattering signals from the two final states, 2*p*_1/2_ and 2*p*_3/2_, and an extension toward the high-energy cutoff edge. Because the cutoff edge represents the maximum outgoing photon energy of the scattering process, the energy difference between the cutoff edge and the *Kα*_1_ maximum Δ*E*_min_ indicates the minimum additional energy required for electron ionization through scattering. Consequently, this cutoff edge is a marker of energy at the lowest vacant level. The *K*-edge energy (*E_K_*_-edge_) was extracted from the HEROS spectrum at the 2*p*_3/2_ level by using the following equation:

$$E_{K-\text{edge}}=\hbar \omega_{\text{in}}+\Delta E_{\text{min}}.$$ (3)

The *E_K_*_-edge_ value derived from our HEROS measurements is determined to be 8980.5 ± 0.6 eV, consistent with the known value.^32^

As Δ*E*_min_ decreased, the HEROS spectra exhibited higher intensities and improved SNR, as shown in Fig. 3(b). The SNR is defined as the ratio of the total fluorescence intensity of an averaged image to the standard deviation of the background noise. The incident photon energies in proximity to the resonance condition are favored for maximizing the HEROS signal intensities. However, when Δ*E*_min_ is excessively small, HEROS spectra unavoidably overlap with *Kα* emissions. Considering that the spectral line shape near the cutoff edge is sensitive to the electron distribution near the Fermi level, this overlap is not ideal. Practically, the XFEL energies must be sufficiently tuned below the absorption edge, adhering to the following conditions to ensure that the cutoff edge is unaffected:

$$\hbar \omega_{\text{in}}<E_{1}-2E_{\text{FWHM}},$$ (4)

where *E*_FWHM_ is the FWHM of a *Kα*_1_ line. In this measurement, the optimal XFEL energy is found to be 8975 eV (Δ*E*_min_ = 5.5 eV), and the XFEL-to-HEROS conversion efficiency at this energy is estimated to be approximately 4%.

**FIG. 4.**
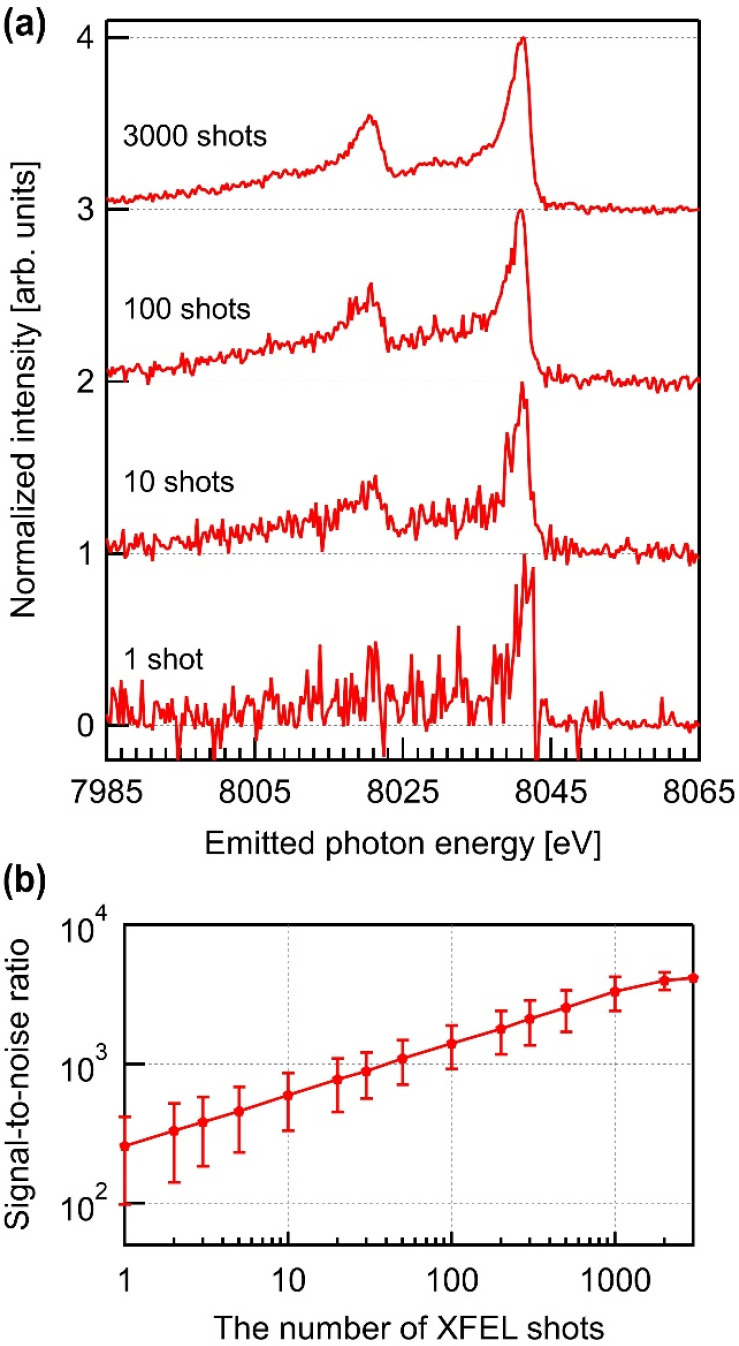
(a) The HEROS spectra and (b) SNRs for various number of XFEL shots. The XFEL photon energy is 8975 eV. The spectra are shown with an offset along the vertical axis for clarity.

Figure 4 shows the HEROS spectra averaged for various numbers of XFEL shots and the corresponding SNRs. The photon energy is fixed at 8975 eV. Even in the single-shot case, the cutoff edge and overall spectral shape were distinguishable. The spectral shape becomes well-defined with ∼10 shots, and the SNR improves proportionally with the number of shots until converging around 2000 shots due to the inherent noise of the instruments. If notable distortions occur in the HEROS spectrum owing to temperature variations during non-equilibrium measurements, they should be captured adequately, even in the single-shot mode. Moreover, minor distortions may be sufficiently captured by accumulating approximately 10–100 shots. Given that the self-seeded pulses at the PAL–XFEL provide approximately six times higher spectral brightness than the SASE pulses, this result indicates that the self-seeding operation overcomes the limitations posed by the low cross sections of scattering events, facilitating the successful application of the HEROS technique in single-shot-based measurements.

### C. Reconstruction of x-ray absorption spectrum from HEROS

**FIG. 5.**
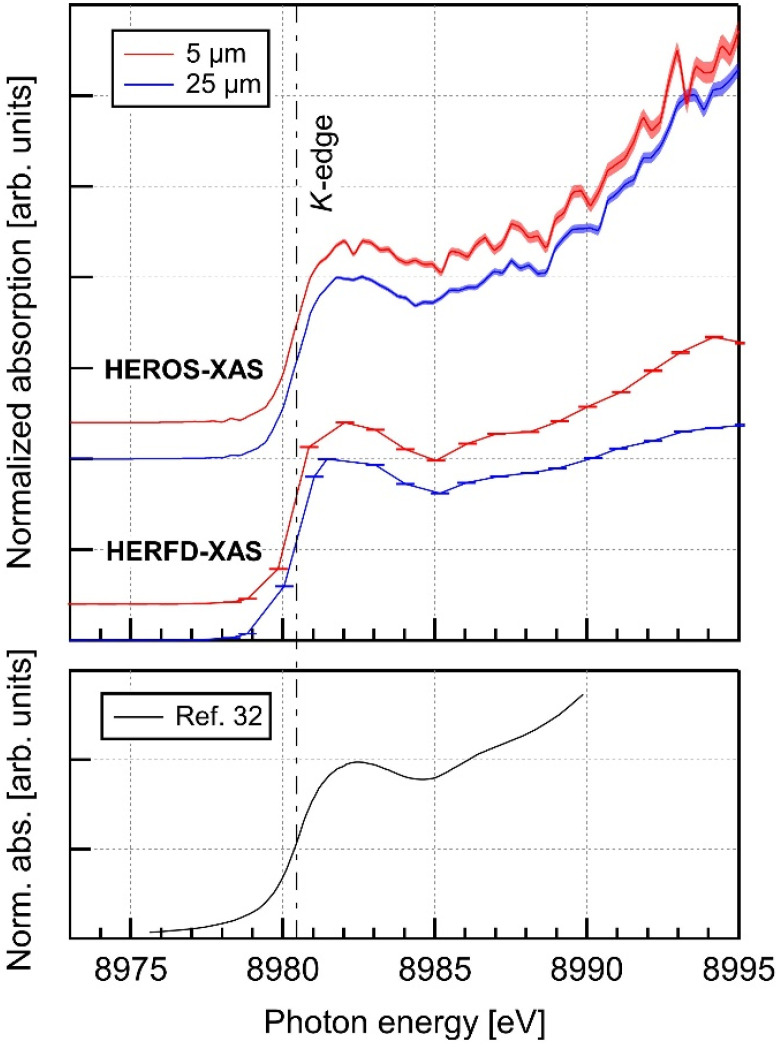
X-ray absorption spectra of 5- (red) and 25-*μ*m-thick (blue) Cu foils obtained by the HEROS and fluorescence-yield measurements, along with the reference spectrum from Ref. 32. The spectra are plotted with a vertical offset for clarity. All spectra are normalized to coincide at the local maxima at 8922 eV.

Figure 5 compares the x-ray absorption spectra of 5- and 25-*μ*m-thick Cu foils obtained using the two methods. The first set of spectra, labeled as HEROS-XAS, was reconstructed using Eq. (2) from the HEROS signals at an incident photon energy of 8975 eV. The level energies and widths were obtained from tabulated data.^32–34^ The second set was derived from *Kα*_1_ fluorescence signals in Fig. 2. The energy resolution of the measurements is smaller than the natural width of the 1*s* level of Cu (∼1.6 eV), characterizing this fluorescence-based technique as high-energy-resolution fluorescence-detected x-ray absorption spectroscopy (HERFD-XAS).^35–37^

Below and near the *K*-absorption edge, both sets of spectra exhibited shapes nearly identical to the reference spectrum. The derivative of HEROS-XAS near the absorption edge has a width of 1.3–1.4 eV, primarily determined by the measurement resolution. This indicates that the HEROS-XAS resolution remains unaffected by inner-shell broadening. Above the edge, the two methods exhibit distinct characteristics. Specifically, HERFD-XAS for the thick foil showed lower absorption at higher energies than that for the thin foil, which was attributed to spectral modulations by the energy-dependent absorption of incident photons by the sample. In contrast, HEROS-XAS yielded consistent results irrespective of foil thickness. The resistance to line broadening and the self-absorption effect make the HEROS technique particularly valuable for obtaining precise information about the electronic structures of samples with unspecified thicknesses or concentrations.

### D. HEROS simulation for warm dense Cu

This section explores the potential application of HEROS measurements for high-temperature matter, achieved by irradiating a solid sample with intense femtosecond optical laser pulses. Energy deposition occurs within femtosecond timescales, inducing an isochoric temperature elevation. Depending on the laser fluence, solid density matter with several eV temperatures, referred to as warm dense matter, can be formed before hydrodynamic expansion is initiated in the picosecond to nanosecond range.^13,38^

**FIG. 6.**
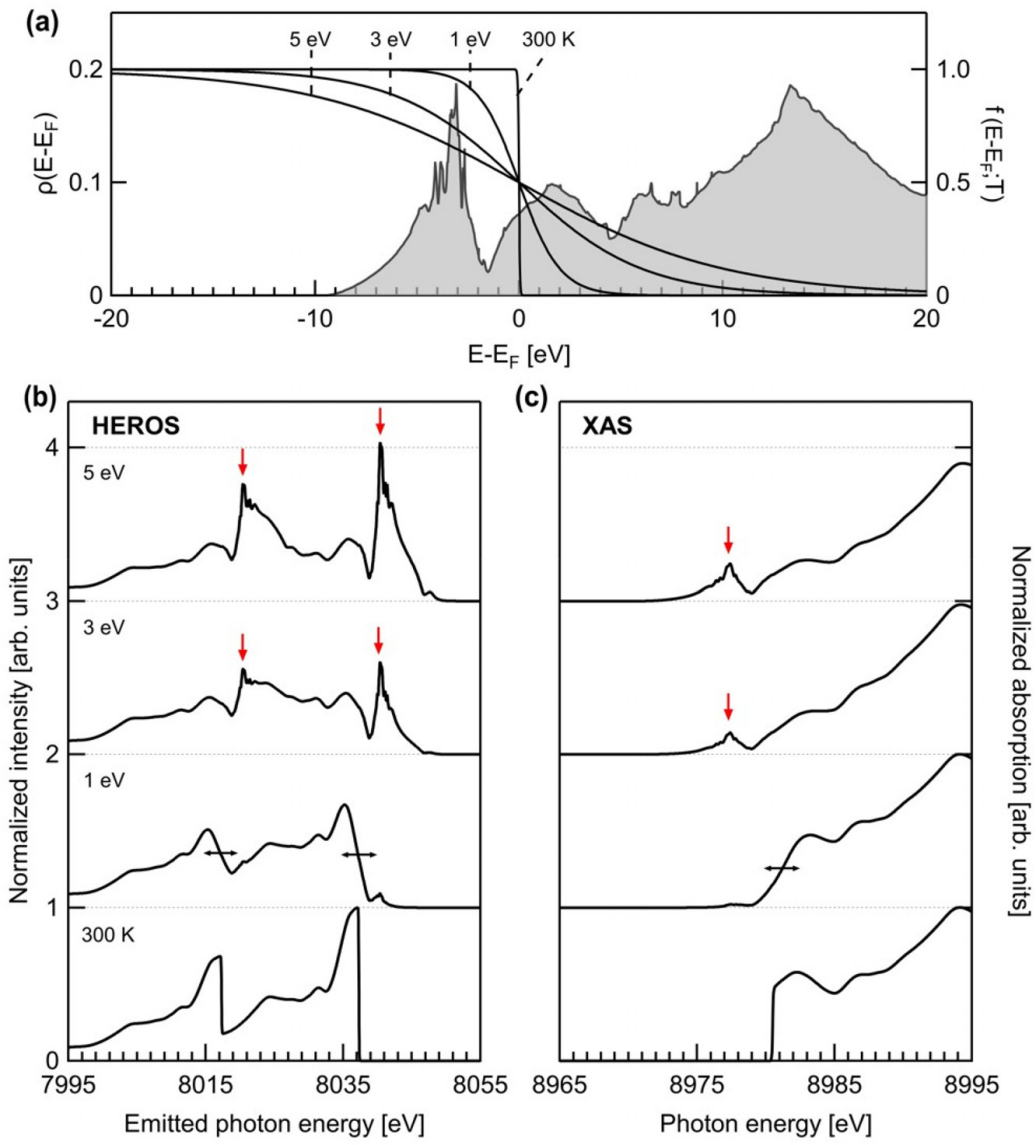
(a) The projected DOS for *p*-orbitals of Cu (left axis) and the Fermi–Dirac distributions with various temperatures (right axis). (b) The simulated HEROS spectra for Cu at various temperatures. (c) The x-ray absorption spectra for the corresponding conditions. Data are drawn with an offset along the vertical axis.

Figure 6(b) shows the calculated HEROS spectra of Cu at various electron temperatures. These spectra were obtained using the projected density of states for *p*-orbitals, as depicted in Fig. 6(a). Density functional theory calculations using the VASP code were employed to evaluate the projected DOS,^39^ considering a plane wave basis set up to a kinetic energy of 600 eV, 30 × 30 × 30 uniform Brillouin zone sampling, Perdew–Burke–Ernzerhof-type generalized gradient approximation, and the projected augmented wave method.^40^ A finite-temperature Fermi distribution of the electrons was applied to each case. The XFEL energy for photoexcitation was set to 8970 eV. At 300 K, the calculated spectra reproduced all critical features observed in Fig. 3(a). As the temperature increased, the calculated HEROS spectra exhibited substantial changes in overall shape. At 1 eV, the sharp edge of HEROS broadened (black arrows), indicating the broadening of the Fermi distribution and opening of channels for core electrons to levels below the *K*-edge. With a further temperature increase, the hole population below the edge increased, resulting in pre-peaks (red arrows) emerging beyond the high-energy cutoff, particularly pronounced at 5 eV.

For comparison, the x-ray absorption spectra at the corresponding temperatures were calculated and are presented in Fig. 6(c). Despite conveying similar information, changes near and below the edge regime were not as strongly observed as those in the HEROS cases. This is because, in HEROS, electrons must acquire energy from radiative decay from the 2*p* levels. As the required energies increase, the scattering probability decreases, resulting in a reduction in the scattering signals as they move toward a lower energy range in the spectrum. The spectral gating is described by the Lorentzian function in Eq. (2), centered at *E*_1_–*E*_2_: This enables HEROS to demonstrate high sensitivity to modifications in unoccupied electronic states around the Fermi level, providing an advantage in investigating the altered electronic structures of warm dense matter systems at temperatures of a few electron volts.

## IV. CONCLUSIONS

This work demonstrates the capability of HEROS measurements with self-seeded pulses at the PAL–XFEL facility. HEROS has emerged as an efficient and precise probing tool with several advantages, including freedom from x-ray energy scanning, core-hole lifetime broadening, and self-absorption effects. Although the relatively low cross section of HEROS might be perceived as a drawback, the unprecedented spectral brightness of the self-seeded XFEL successfully circumvented this limitation, enabling single-shot measurements. Notably, HEROS exhibits the distinctive capability of accentuating information on electronic structures near the Fermi level. These advantages make the HEROS technique at an XFEL facility a valuable tool for scrutinizing ultrafast dynamics in diverse materials and conditions, including warm dense matter and non-equilibrium research.

## Data Availability

The data that support the findings of this study are available from the corresponding author upon reasonable request.
